# Neural dynamics of predictive timing and motor engagement in music listening

**DOI:** 10.1126/sciadv.adi2525

**Published:** 2024-03-06

**Authors:** Arnaud Zalta, Edward W. Large, Daniele Schön, Benjamin Morillon

**Affiliations:** ^1^ Aix Marseille Université, Inserm, INS, Institut de Neurosciences des Systèmes, Marseille, France.; ^2^ APHM, INSERM, Inst Neurosci Syst, Service de Pharmacologie Clinique et Pharmacovigilance, Aix Marseille Université, Marseille, France.; ^3^ Department of Psychological Sciences, Ecological Psychology Division, University of Connecticut, Storrs, CT, USA.; ^4^ Department of Physics, University of Connecticut, Storrs, CT, USA.

## Abstract

Why do humans spontaneously dance to music? To test the hypothesis that motor dynamics reflect predictive timing during music listening, we created melodies with varying degrees of rhythmic predictability (syncopation) and asked participants to rate their wanting-to-move (groove) experience. Degree of syncopation and groove ratings are quadratically correlated. Magnetoencephalography data showed that, while auditory regions track the rhythm of melodies, beat-related 2-hertz activity and neural dynamics at delta (1.4 hertz) and beta (20 to 30 hertz) rates in the dorsal auditory pathway code for the experience of groove. Critically, the left sensorimotor cortex coordinates these groove-related delta and beta activities. These findings align with the predictions of a neurodynamic model, suggesting that oscillatory motor engagement during music listening reflects predictive timing and is effected by interaction of neural dynamics along the dorsal auditory pathway.

## Teaser

Interacting neural dynamics along the dorsal auditory pathway effect the experience of groove during music listening.

## INTRODUCTION

Dancing to the beat of music is universal (*1*, *2*). However, it is also distinctly specific, being exclusive to the auditory modality, musical stimuli, and certain music. Music is most often considered as an auditory phenomenon, but, under an ecological and phylogenetic perspective, it is tightly coupled to dance (*3*, *4*). Dance requires synchronizing body movements with the musical rhythm via audio-motor interactions (*5*–*8*). Not all music induces dance equally, but why does it sometimes urge us to dance? What are the brain mechanisms supporting the musical wanting-to-move experience called groove (*9*–*15*)?

The sensation of groove engages motor and reward networks (*13*, *16*, *17*). Even in the absence of physical movement, the perception of temporally structured musical rhythms triggers activity in motor regions, encompassing premotor cortices, supplementary motor areas, and the basal ganglia (*18*–*21*). This motor activity has been associated with the neural recycling of action circuits for time estimation (*22*–*26*). This is related to the framework of active sensing (*27*–*30*). Active sensing refers to the fact that perception is strongly shaped by motor activity, which notably imposes temporal constraints on the sampling of sensory information, particularly in the delta band. This frequency corresponds to the time constant of motor-related neural dynamics (*31*–*33*), which is conserved across species (*34*) and reflected in the range of natural movements and of music (*35*).

Accordingly, during auditory perception, the motor system encodes temporal predictions information and can optimize auditory processing (*7*, *36*–*39*). Recently, a Bayesian model of groove has been proposed, in which the experience of groove is related to predictive timing and more precisely correlates with the precision-weighted temporal prediction error computation during the processing of musical rhythms (*5*, *40*).

However, the neurophysiology of groove is still unclear. For instance, it remains uncertain whether groove ratings correlate with the neural cortical activity that tracks the beat (*41*, *42*), with intrinsic motor-related neural dynamics (*43*), or how the transition from perceiving musical rhythms to motor engagement unfolds. Neurodynamic models offer a computationally rigorous and neurophysiologically plausible approach to understanding the emergence of music-related cognitive phenomena (*44*), being more closely informed by neural dynamics than Bayesian models (*45*). By combining a neurodynamic model (*44*, *46*) with the concept of auditory active sensing (*37*, *47*), we propose a canonical dynamical framework to understand the spontaneous emergence of movements during music listening. We hypothesize that manipulating the rhythmic properties of music suffice to induce a covert motor engagement during music listening, via changes in audio-motor neural dynamics.

## RESULTS

**Fig. 1.**
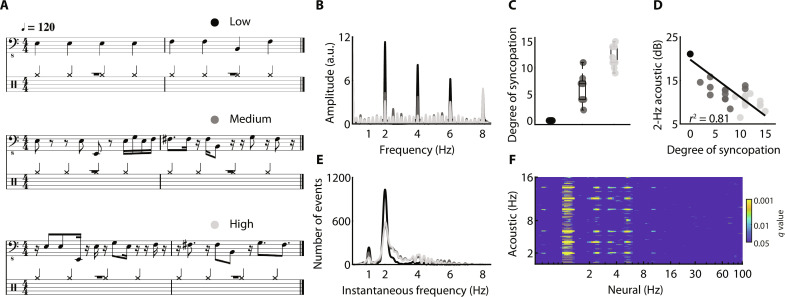
Stimulus set. Twelve 8-s melodies with a 2-Hz beat were created. For each melody, three variants were designed to vary the level of rhythmic predictability (degree of syncopation) while minimizing other acoustic variations. (**A**) Example of a melody with a low (black), medium (gray), or high (light gray) degree of syncopation. (**B**) Averaged modulation spectrum of the acoustic temporal envelope of the melodies, for each of the three conditions. a.u., arbitrary units. (**C**) Degree of syncopation of the melodies, grouped by condition. Each dot represents one melody. (**D**) Amplitude of the acoustic envelope at 2 Hz (in decibels; “2-Hz acoustic”), as a function of the degree of syncopation, across melodies. Data were approximated with a linear function. Pearson’s *r*^2^ is reported. Shades of gray indicate the conditions. (**E**) Behavioral tapping experiment: distribution of the instantaneous frequency of finger tapping per condition, cumulated across melodies and participants, recorded while participants were reproducing the rhythm of their dance step while listening to the melodies. (**F**) MEG experiment: Statistical map of neural coding of the acoustic temporal modulation spectrum, from the power spectrum of the whole-brain MEG signals recorded while participants were listening to melodies (*q* < 0.05, FDR-corrected).

We created a stimulus set of 12 short melodies with a 2-Hz beat. To vary their level of rhythmic predictability, three variants were derived from each melody, using an ascending degree of syncopation (low, medium, high; Fig. 1, A to C). As expected, the degree of syncopation was inversely proportional to the amplitude of the acoustic dynamics at 2 Hz [*coefficient of determination *r**^2^(34) = 0.81, *P* < 0.001; Fig. 1D]. Nonetheless, when asked to reproduce the rhythm of their dance step while listening to the melodies, a first group of participants (*n* = 14) predominantly moved at the 2-Hz beat across conditions (Fig. 1E), confirming that, when listening to music, movements tend to be synchronized to the beat, the most salient rhythmic event (*21*, *48*, *49*). Next, in a second experiment, we recorded magnetoencephalography (MEG) data while participants (*n* = 29) listened to the melodies. A first multivariate pattern (decoding) analysis on channel-level MEG data showed an absence of 1:1 mapping between the acoustic temporal envelope and neural frequencies. Instead, acoustic dynamics are decoded at 2 Hz and, to a lesser extent, at its harmonics [*q* < 0.05, false discovery rate (FDR)–corrected; Fig. 1F; see Materials and Methods). Thus, both behavioral motor and neural cortical dynamics principally track the 2-Hz beat.

**Fig. 2.**
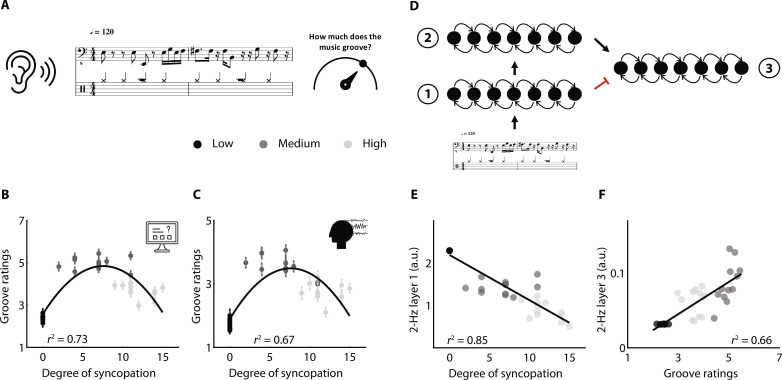
Behavioral experiments and neurodynamic model. (**A**) Main experimental design: In two experiments (online and MEG), participants listened to melodies. After each melody, they were asked to rate it in terms of groove, defined as the extent to which they wanted to move to the music. (**B** and **C**) Behavioral rating of participants (groove) acquired (B) online (from 1 to 7) or (C) during the MEG experiment (from 1 to 5), as a function of the degree of syncopation, across melodies. Data were approximated with a quadratic function. Adjusted *r*^2^ is reported. Shades of gray indicate the conditions. Error bars indicate SEM. (**D**) Neurodynamic model: Each layer represents a network of coupled oscillators at different frequencies. The rhythm of melodies was input in the first layer. Arrows represent the coupling across layers (black is excitatory, and red is inhibitory). (**E**) Amplitude of the output of layer 1 at 2 Hz, as a function of the degree of syncopation, across melodies. Data were approximated with a linear function. Pearson’s *r*^2^ is reported. Shades of gray indicate the conditions. (**F**) Amplitude of the output of layer 3 at 2 Hz, as a function of groove ratings (from the online experiment), across melodies. Same conventions as in (E).

We also asked the participants to rate the groove for each melody (Fig. 2A). Participants’ desire to move highly correlates with the degree of syncopation but in a nonlinear manner. Furthermore, we replicated these results in a third group of participants (*n* = 66) performing the experiment online (Fig. 2, B and C; see Supplementary Results). This inverse U-shape profile is well approximated with a quadratic function [online experiment: adjusted *r*^2^(33) = 0.73; MEG experiment: adjusted *r*^2^(33) = 0.67], confirming previous findings of moderately syncopated melodies inducing strongest wanting-to-move experiences (*12*, *14*, *15*, *50*, *51*). This behavior shows that motor engagement indexes neither temporal predictability (highest in the low-syncopated condition) nor temporal prediction errors (highest in the high-syncopated condition) but is compatible with the notion of a precision-weighted temporal prediction error computation (*5*, *40*).

Next, we created a neural network model composed of three layers, each layer representing a network of oscillators spanning a range of frequencies (*44*, *52*, *53*). Following previous modeling work on the perception of rhythmic pulse (*44*), the melodies’ rhythms were presented as input to a first network layer (layer 1), which modeled auditory cortical dynamics as an oscillatory network operating near a Hopf bifurcation, and two other network layers (layers 2 and 3), which modeled motor cortical dynamics operating near a double limit cycle bifurcation (Fig. 2D; see Materials and Methods). This three-layer neurodynamic model accounts for the nonlinear transformation from a syncopated stimulus rhythm to the subjective experience of groove. We observed a dissociation between (i) a strong linear correlation of the degree of syncopation with the 2-Hz activity in layer 1 [*r*^2^(34) = 0.85, *P* < 0.001; Fig. 2E) and (ii) a strong linear correlation of groove ratings with the 2-Hz activity in layer 3 [online experiment: *r*^2^(34) = 0.66, *P* < 0.001; Fig. 2F], with far less contributions of the other layers (see Supplementary Results and fig. S1).

**Fig. 3.**
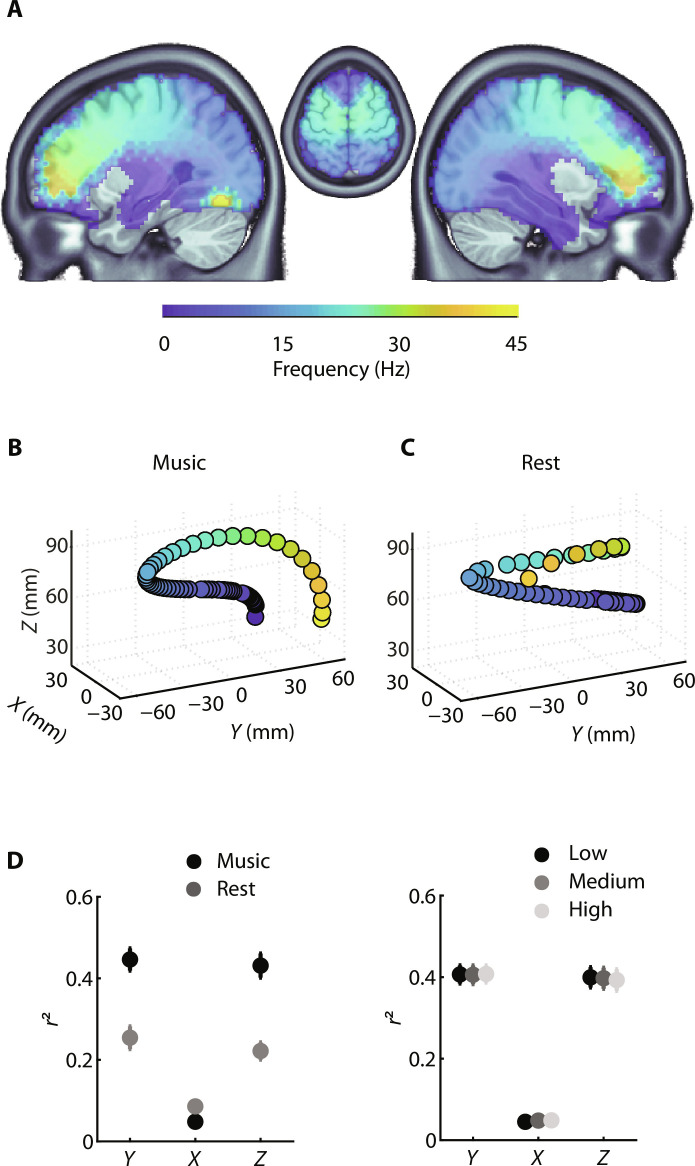
Spectral gradient of neural activity along the dorsal auditory pathways during music listening. (**A**) Dominant frequency across the brain volume during music listening of the melodies (1 to 45 Hz; after removal of the 1/*f* decay of the neural power spectrum). (**B**) Data were approximated at the group level with a polynomial function, independently for each dimension (*X*, *Y*, and *Z*) of the MNI space. (**C**) Control analysis: Same as in (B) but from a resting-state MEG dataset. (**D**) Comparison of the quality of fits (*r*^2^), estimated at the individual level, between (left) the music and rest MEG datasets and (right) the three melodic conditions. Error bars indicate SEM.

We next analyzed the neural dynamics of cortical activity while listening to the melodies. We first estimated the 1/*f*-rectified power spectrum (1 to 100 Hz; see Materials and Methods) of neural activity at the source level and observed that the spatial and spectral dimensions are closely related, in the form of a bilateral spectral gradient along the dorsal auditory pathways (Fig. 3A). The frequency of the dominant activity progressively increases from auditory regions (<10 Hz) to the motor cortex (20 to 30 Hz), up to the inferior frontal cortex (>30 Hz; activity > 45 Hz was not observed).

We quantified this spectro-spatial relationship by fitting the dominant frequency of each vertex to each spatial dimension (*x*, *y*, and *z*) and confirmed that this gradient travels conjointly along the antero-posterior (*Y*) and ventro-dorsal (*Z*) dimensions, compatible with the localization of the dorsal auditory pathways (Fig. 3B). To investigate whether this spectral gradient is specific to music listening or reflects a more generic neurophysiological signature of brain dynamics, i.e., with intrinsic timescales exhibiting a spatial gradient (*54*, *55*) along the dorsal auditory pathways, we performed the same analysis on resting-state data acquired on the same participants (*n* = 29). We failed to observe a close relation between spectral and spatial dimensions, as indexed by the much less spatially structured pattern of the spectral gradient (Fig. 3C). This dynamic reorganization in the form of a spectral gradient along the dorsal auditory pathways during music listening was confirmed by an individual level estimation of the quality of fit for the two datasets [repeated-measures analysis of variance (rm-ANOVA): main effect of dataset: *F*_1,28_ = 54.4, *P* < 0.001; Fig. 3D, left].

Further analyses suggested that such spectral gradient is a general characteristic of music listening, independent of the specific acoustic, melodic, or cognitive attributes of the music. We observed that the spectral gradient does not vary across conditions (low, medium, and high), neither in shape (fig. S2A) nor in its quality of fits (Fig. 3D, right; rm-ANOVA: main effect of condition: *F*_2,56_ = 0.1, *P* = 0.9). This latter result was robust even at the level of individual melodies (fig. S2B; rm-ANOVA: main effect of melodies: *F*_35,980_ = 0.8, *P* = 0.7).

**Fig. 4.**
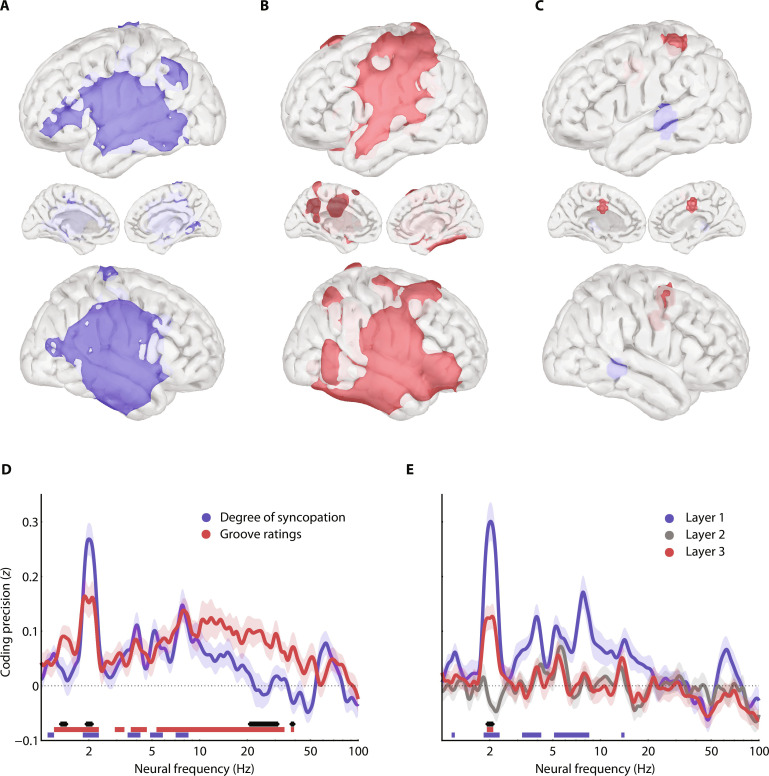
Spatial and spectral coding of degree of syncopation and groove ratings. (**A** to **C**) Spatial map of significant neural coding of (A) the degree of syncopation (blue) and (B) groove ratings (red), from the power spectrum (1 to 100 Hz) of neural data recorded while participants were listening to melodies. Significant results reported at *q* < 0.005, FDR-corrected. (C) Spatial map of the difference in coding precision between degree of syncopation and groove ratings. Blue (or red) indicates preferred coding for the degree of syncopation (or groove ratings; *P* < 0.005, uncorrected). (**D**) Spectrum of neural coding of the degree of syncopation (blue) and groove ratings (red), from whole-brain MEG signals. Blue and red horizontal lines indicate frequencies with significant coding values (*q* < 0.005, FDR-corrected) for degree of syncopation and groove ratings, respectively. The black line indicates frequencies with significant differences in coding precision between degree of syncopation and groove ratings (*q* < 0.05, FDR-corrected). Error bars indicate SEM. (**E**) Spectrum of neural coding of the 2-Hz amplitude of layer 1 (blue), layer 2 (gray), and layer 3 (red) of the neurodynamic model, from whole-brain MEG signals. Red and blue horizontal lines indicate frequencies with significant coding values for layers 1 and 3 (*q* < 0.005, FDR-corrected). The black line indicates frequencies with significant differences in coding precision between layers 1 and 3 (*q* < 0.05, FDR-corrected). Error bars indicate SEM.

Next, to investigate the neural correlates of the degree of syncopation and groove ratings across spatial or spectral dimensions, we conducted multivariate pattern (decoding) analyses on the MEG data at either the source level (Fig. 4, A to C) or the channel level (Fig. 4D; see Materials and Methods). Moreover, in each dimension, we computed the difference in coding precision between degree of syncopation and groove ratings, to investigate their selective neural underpinning (Fig. 4, C and D). These analyses first revealed that both the degree of syncopation and groove ratings are primarily coded in the bilateral and surrounding auditory regions (Fig. 4, A and B; *q* < 0.005, FDR-corrected) and in beat-related 2-Hz neural dynamics (Fig. 4D; *q* < 0.005, FDR-corrected), this latter result validating the key prediction of the neurodynamic model. This neural pattern significantly preferentially codes for the degree of syncopation (Fig. 4C, spatial analysis, *P* < 0.005, uncorrected; Fig. 4D, spectral analysis, *q* < 0.05, FDR-corrected). By contrast, groove is better decoded in the left parietal, supplementary motor, and right motor cortex (Fig. 4C, spatial analysis, *p* < 0.005, uncorrected) and at low-delta (1.3 to 1.5 Hz) and beta (20 to 31 Hz and 38 to 39 Hz) neural dynamics (Fig. 4D, spectral analysis, *q* < 0.05, FDR-corrected). A complementary region-of-interest (ROI) analysis (estimated from Fig. 4E) confirmed that the degree of syncopation is significantly coded only in bilateral auditory regions at 2 Hz (and harmonics) and that the experience of groove is preferentially coded in (left-lateralized) auditory and parietal delta dynamics and in (alpha) beta neural dynamics (fig. S3; *q* < 0.05, FDR-corrected; see Materials and Methods).

Additional analyses show that the 2-Hz amplitude in layers 1 and 3 of the neurodynamic model, which, respectively, exhibit strong linear correlations with the degree of syncopation and groove ratings (as depicted in Fig. 2, E and F), are significantly coded in 2-Hz neural dynamics (and harmonics; Fig. 4E; *q* < 0.005, FDR-corrected; neural coding of layer 2 amplitude does not reach statistical significance). However, delta (1.4 Hz) and beta (20 to 30 Hz) neural dynamics, which also coded for groove ratings, are conspicuously absent from the predictions of the neurodynamic model (notably layer 3; Fig. 4, D versus E).

**Fig. 5.**
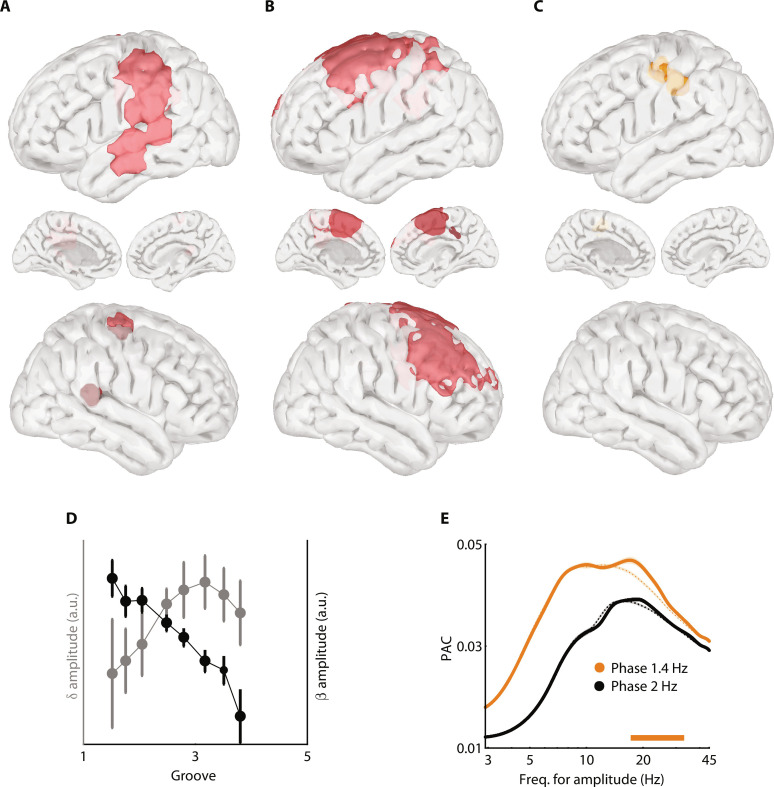
Groove-related neural dynamics in the dorsal auditory pathway. (**A**) Spatial map of neural coding of groove ratings from delta (1.3 to 1.5 Hz) neural activity. (**B**) Spatial map of neural coding of groove ratings from beta (20 to 30 Hz) neural activity. (**C**) Spatial map of significant local phase-amplitude coupling (PAC) between delta (1.3 to 1.5 Hz) phase and any amplitude frequency (3 to 45 Hz). (**D**) Amplitude of delta (1.3 to 1.5 Hz; gray) and beta (20 to 30 Hz; black) neural activity as a function of groove ratings, in the significant PAC cluster [from (C)]. Error bars indicate SEM. (**E**) Details of PAC estimates in the significant PAC cluster [from (C); plain lines] or averaged across whole brain (dashed lines), between 1.3- and 1.5-Hz (orange) or 2-Hz (beat frequency; black) phase and any amplitude frequency (3 to 45 Hz). The orange horizontal line indicates a significant increase of PAC in the inset cluster compared to whole brain. (A to E) Significant results reported at *q* < 0.005, FDR-corrected.

To further investigate the neural coding of groove ratings beyond the predictions of the model, we computed decoding analyses on source-level MEG data focusing on beat-related 2-Hz (fig. S4), delta (1.3 to 1.5 Hz; Fig. 5A), and beta (20 to 30 Hz; Fig. 5B) neural dynamics. This coding of groove ratings is adjusted to the spectral gradient of activity along the dorsal auditory pathway (Fig. 3, A and B): Groove-related delta and beta activities are visible respectively along the inferior portion of the dorsal auditory pathway bilaterally (Fig. 5A; *q* < 0.005, FDR-corrected) and in dorsal, premotor regions (Fig. 5B; *q* < 0.005, FDR-corrected). Critically, the left sensorimotor cortex couples groove-related delta and beta activities through phase-amplitude coupling (Fig. 5C; *q* < 0.005, FDR-corrected) during music listening and the reported coupling is specific to low-delta (1.3 to 1.5 Hz) and beta (20 to 30 Hz) dynamics and is not significant for beat-related 2-Hz activity (Fig. 5E). Moreover, within this region, delta activity increases, while beta amplitude monotonically decreases with groove ratings (Fig. 5D). This observation aligns with the whole-brain decoding results (Figs. 4D and 5, A and B) and suggests that the experience of groove is reflected in amplitude fluctuations in the dorsal auditory pathway. These analyses evidence the distributed neural dynamics implicated in the nonlinear transformation from a syncopated stimulus rhythm to the subjective experience of groove.

## DISCUSSION

Why do humans spontaneously want to move to music? Here, we characterize the computational and neurophysiological bases of this phenomenon by demonstrating that (i) behavioral motor and neural cortical dynamics principally track the beat of music (Fig. 1, E and F); (ii) the pleasurable wanting to move to music, the experience of groove, depends on the temporal regularities present in the music (the degree of syncopation; Fig. 2, B and C); (iii) this phenomenon is accounted for by a neurodynamic model and may be described in terms of the timing of auditory events relative to expectancies embodied in oscillations (Fig. 2, D to F); (iv) an ascending spectral gradient along the dorsal auditory pathways emerges during music listening (Fig. 3, A to D); (v) the dorsal auditory pathways contribute to the coding of the degree of syncopation in auditory regions and of the experience of groove more dorsally, selectively in low-delta (1.3 to 1.5 Hz) and beta (20 to 30 Hz) neural dynamics (Figs. 4, A to D, and 5, A and B); and (vi) these groove-related delta-beta neural dynamics are coupled in the left sensorimotor cortex (Fig. 5, C to E).

These results extend seminal studies on the quadratic relationship between rhythmic predictability and the experience of groove (*9*, *10*), which we replicated with an original set of melodies, in two independent experiments (see Materials and Methods). This relationship has been explained using Bayesian inference, with groove arising when precise temporal priors are violated by sensory evidence, here recurring syncopated rhythmic patterns (*5*, *56*). Here, we provide an alternative dynamical system account (*44*). Mode locking of neural oscillations to complex rhythms allows the emergence of neural resonance at metrical frequencies. Our dynamical model shows that the experience of groove can be parsimoniously explained as embodied resonance to a beat and result from the combination of excitatory and inhibitory inputs from two successive layers. Our MEG results further suggest that this is implemented along the dorsal auditory pathway, which connects the auditory and motor dynamics. The Bayesian and dynamical models converge in the sense that groove may be described as affordance for movements that interact with the stimulus temporal structure, relying on the timing of auditory events relative to expectancies. The neurodynamic model further specifies temporal expectations as being the consequence of neural resonance, i.e., being embodied in oscillations, and hence provides a physiologically plausible model to predictive timing (*7*, *24*, *25*, *47*) and the experience of groove (*5*, *15*).

The first two network layers were intended to reflect the auditory rhythm (auditory cortex) and the perception of pulse and meter (motor planning cortex) (*44*). Oscillations in layer 2 may be considered as temporal predictions, i.e., expectations about the timing of rhythmic events (*57*, *58*). Critically, layer 2 activity arise not based on a learned model but based on mode locking of neural oscillators to other frequencies in the rhythm. To these, we added a third layer that received inhibitory connections from layer 2 and excitatory connections from layer 1 (see Materials and Methods). Hence, layer 3 responds to the difference between the time-dependent oscillations of layer 2 (pulse per meter) and layer 1 (auditory rhythm) and can thus be interpreted as reflecting the divergence between temporal predictions (layer 2) and the actual input (layer 1). That groove ratings only correlate with 2-Hz amplitude in layer 3 indicates that the groove phenomenon may be described in terms of the timing of auditory events (layer 1) relative to expectancies (layer 2) embodied in oscillations.

The neurodynamic model was not implemented to reflect the specificity of different cortical areas regarding their cyto- and myelo-architectonic structure, local and long-range connectivity, activity, or function (*59*). In particular, it did not carry any information regarding the intrinsic rhythmic neural activity characteristic of auditory and motor areas (*31*, *32*, *60*, *61*). In light of this, it is notable that layer 3, which captures groove ratings, is specifically coded in beat-related 2-Hz neural activity but not in low-delta (1.4 Hz) and beta (20 to 30 Hz) dynamics. The wanting-to-move (groove) is a multidimensional phenomenon (*10*), hypothesized to be affected by a variety of cognitive processes such as temporal predictions (“inner representation of temporal regularity”), temporal attention (“time-related interest”), musical pleasure, and arousal (*62*). The neural underpinnings of these cognitive processes are likely partially reflected in distinct neural dynamics, such as delta (1.4 Hz), beat-related 2-Hz, and beta frequencies. The neurodynamic model precisely captures the groove-related variance exclusively coded within the 2-Hz neural activity, possibly related to temporal predictions of the 2-Hz beat. A model capturing the other cognitive dimensions of groove, associated with delta (1.4 Hz) and beta dynamics, remains to be developed.

That the motor system contributes to auditory perception is also supported by human psychophysics and neuroimaging data, particularly when listening to rhythmic auditory streams (*20*, *21*, *37*, *39*). Here, we extend these findings to an ecological music listening situation and further reveal that activity in the dorsal auditory pathway is organized in the form of a spectral gradient. This anatomo-spectral gradient could be instrumental in structuring the information flow. Our finding extends a few recent reports of the presence of a spectral gradient along different cortical pathways (*54*, *55*, *63*, *64*) and provide clear evidence of a tight relation between the anatomical and dynamical dimensions of the brain. Whether this gradient acts as a support for information transfer, directly codes for music-specific information, or is constitutive of auditory perception at large remains to be investigated, but our results indicate that it does not code for the sensory (degree of syncopation) or cognitive (groove ratings) investigated variables.

During music listening, this gradient is organized around the left sensorimotor cortex that is implicated in feedback signaling during auditory perception (*37*, *65*). This region might correspond to area 55b, a proposed keystone of sensorimotor integration critical in both music (*66*) and speech (*67*, *68*) perception. This area contributes to the emergence of audio-motor coupling and plays a role in coordinating ventral delta (1.4 Hz) and dorsal beta (20 to 30 Hz) neural dynamics. These dynamics were not present in the stimulus and instead reflect intrinsic cortical dynamics. While beta dynamics (∼12 to 30 Hz) are typical of sensorimotor areas and associated with movement planning and execution (*69*), low (<20 Hz) and high (20–30 Hz) beta bands stem from distinct neurophysiological origins [e.g., (*70*)] and, respectively, predominate in the primary motor cortex and associative motor areas [notably the premotor cortex; (*71*)]. Our findings of groove ratings being preferentially coded in premotor high beta (20 to 30 Hz) dynamics are well in line with its reported role in temporal predictions and dynamic attention (*24*, *37*, *38*, *71*, *72*). The groove-related delta (1.4 Hz) activity observed in the dorsal auditory pathway corresponds to the optimal rate for auditory temporal predictions. This timescale characterizes the capacity for auditory temporal attention (*36*), a function associated with the left parietal cortex (*73*). This finding notably supports a model that considers neuronal oscillations as intrinsic dynamical mechanisms capable of embodying neural computations (*45*, *74*). The reported delta-beta coupling could reflect the temporal alignment between temporal predictions and attention fluctuations (*39*, *47*, *75*). Our results further show that the spectral gradient is not spatially reorganized between melodies, while the amplitude of delta and beta dynamics fluctuates with groove ratings. Hence, the experience of groove is probably reflected in amplitude fluctuations within a stable spectral gradient of activity. In conclusion, we show that interacting neural dynamics along the dorsal auditory pathway correlate with the spontaneous emergence of the pleasurable wanting to move during music listening.

## MATERIALS AND METHODS

### Participants and stimuli

#### Participants

A total of 66, 30, and 15 participants (age range, 19 to 71 years; 77% females) were recruited for the online, MEG, and control tapping experiments, respectively. The number of participants was determined as follows. The online experiment was accessible for 2 weeks, with no stopping rule. The MEG experiment followed the guidelines of our MEG center, balancing data collection costs with statistical power. Last, the participant count for the control tapping experiment matched that of a prior similar-task study (*36*). All experiments followed the local ethics guidelines from Aix-Marseille University. Informed consent was obtained from all participants before the experiments. All had normal audition and vision and reported no history of neurological or psychiatric disorders. We did not select participants on the basis of musical or dance training and a short survey made at the end of the experiment informed us that none of them were professional musicians. Participants were financially compensated for their time during the MEG experiment.

#### Acoustic stimuli

A professional musician first composed in MIDI (Musical Instrument Digital Interface) format 12 original melodies lasting 8 s each (stimuli are available on github.com/DCP-INS/Groove). All had the same strictly periodic drum beat at 2 Hz combined with a specific bass melody. To vary the level of rhythmic predictability while minimizing other acoustic variations, two types of variants were derived from each of these 12 original melodies, by varying the degree of syncopation (the amount of syncopation is inversely proportional to the rhythmic predictability). The 12 original melodies constituted the condition of medium degree of syncopation. The condition of low syncopation was created by synchronizing every bass note to the periodic drumbeat, resulting in 12 melodies having a degree of syncopation of 0. The condition of high syncopation was created by maximizing the number of syncopations present in each melody. Syncopation was defined as the appearance of a beat on a metrically weak accent preceding a rest on a metrically strong accent and quantified after Longuet-Higgins and Lee (*76*). The bass melodies were maximally matched between the three variants of the same original melody, with the number of notes per measure, their pitch, and their order being identical or closely matched. Other musical characteristics (volume, timbre, etc.) were kept constant. This procedure resulted in 36 melodies, with degrees of syncopation ranging from 0 to 15, and divided in three conditions, reflecting a low (black), medium (gray), or high (light gray) degree of syncopation for each melody (Fig. 1). Last, the songs were recorded in stereo with a sampling rate of 48 kHz and a bit depth of 24 bit.

### Experimental designs and data acquisition

#### Experimental design of the online experiment

Participants were invited to visit a web page to take part in the survey, hosted by PsyToolkit. After completing the questionnaire, participants were invited to start the experiment. They were first prompted to use headphones or earphones and given the opportunity to pre-evaluate the output volume to adjust them comfortably. Then, the experiment consisted of a listening task in which each of the 36 melodies was presented binaurally once to participants, in a randomized manner. After stimulus offset, participants reported on a keyboard the associated level of groove, defined as the extent to which they wanted to move to this music (*9*–*15*). They had 60 s to answer. A Likert scale between 1 and 7 was used. Instructions were visually displayed on a mid-gray background on a screen computer. During each trial, participants had to fixate a cross, located at the center of the screen. The online experiment lasted ~10 to 15 min.

#### Experimental design of the control tapping experiment

The experiment was performed in the laboratory, using the Psychophysics-3 Toolbox and additional custom scripts written for MATLAB (The MathWorks). Trials consisted of a tapping task in which participants were asked to reproduce, with their indexes on the computer keyboard, the rhythm of the dance step that they would naturally produce when listening to the melodies. Each of the 36 melodies was presented binaurally once to participants, in a randomized manner, at a comfortable hearing level via headphones. Instructions were visually displayed on a mid-gray background on the screen laptop situated at a viewing distance of around 50 cm. On each trial, participants had to fixate a cross, located at the center of the screen.

#### Experimental design of the MEG experiment

The experiment consisted of a listening task in which melodies were presented binaurally to participants, in a randomized manner. Participants were requested to stay completely still while they were listening to the melodies. Each original melody was duplicated, while maintaining the beat structure, which resulted in 16-s-long melodies. This allowed us to optimize the signal-to-noise ratio of the MEG response. Moreover, the experiment was composed of four blocs. In each bloc, each of the 36 duplicated melodies was presented binaurally once to participants, in a randomized manner (144 trials in total). After stimulus offset, participants had 4 s to report on a keyboard the associated level of groove on a Likert scale between 1 and 5. The experiment lasted ~48 min. Of note, we adjusted the scale and duration of responses between online and MEG experiments, due to the limited number of keys on the MEG system’s response box, and to improve comfort and maintain participant concentration throughout the MEG experiment.

#### Experimental design of the resting state experiment

Participants performed two 4-min eyes-open resting-state sessions, at the beginning and at the end of the MEG experiment. The two sessions were pooled for subsequent analyses.

#### MEG data acquisition

MEG data were acquired at the Epileptology and Cerebral Rhythmology Unit from the La Timone hospital, APHM, Marseille (France), using a 4-D Neuroimaging 3600 whole-head system (4-D Neuroimaging, San Diego, CA, USA) composed of 248 magnetometers; at a sampling frequency of 2034.51-Hz electrooculogram and electrocardiogram channels, one audio and five response buttons (LUMItouch optical response keypad) were recorded simultaneously and synchronized with the MEG signal. Presentation software was used for stimulus delivery and experimental control during MEG acquisition. Auditory stimuli were presented binaurally at a comfortable hearing level through insert earphones (E-A-RTONE 3A, Aero Company). Participants were comfortably reclined in the MEG scanner, so that they were physically constrained in their movement and muscularly relaxed. Instructions were visually displayed on a mid-gray background on a screen computer situated at a viewing distance of around 50 cm. On each trial participants had to fixate a cross, located at the center of the screen, to get a visual constant stimulation. Location of the participant’s head with respect to the MEG sensors was recorded both at the beginning and end of each session to potentially exclude sessions and/or participants with large head movements. However, none of the participants moved >3 mm during all sessions.

#### MRI data acquisition

For volume MEG source analysis (i.e., the projection of the MEG sensor data onto the full brain volume), a T1-weighted magnetic resonance imaging (MRI) acquisition of the brain was obtained from each participant (1-mm isotropic voxel resolution).

### Data analyses

#### Timing of motor acts in the control tapping experiment

One participant was excluded from subsequent analyses (hence, *n* = 14) as we failed to detect proper tapping responses. To investigate the dynamics of occurrence of the motor events *m* produced at time *t* (in seconds) by participants during the listening of the melodies, we estimated the instantaneous frequency *F*(*t*) (in hertz) of their finger taps by computing the inverse of the inter-tap interval, given by

$$F\left(t\right)=\frac{1}{m_{t}-m_{t-1}}$$

#### Spectral decomposition of the acoustic stimuli

To estimate the temporal envelope of each melody, the sound signal was decomposed into 32 narrow frequency bands using a cochlear model, and the absolute value of the Hilbert transform was computed for each of these narrowband signals. The broadband temporal envelope resulted from the summation of these absolute values and was used as the acoustic signal for all subsequent analyses. Using a fast Fourier transform, we then decomposed the acoustic signal of each melody from 1 to 9 Hz, to obtain the acoustic temporal modulation spectrum (i.e., the spectrum of the temporal envelope).

#### MEG data preprocessing

Preprocessing was performed with Brainstorm (*77*), following the good practice guidelines (*78*). Briefly, we removed electrical artifacts using notch filters (at 50 Hz and its first three harmonics), slow drifts using high-pass filtering (at 0.3 Hz), and eye blink and heartbeat artifacts using source signal projections. Data were split into 20-s trials, from −2 to +18 s relative to stimulus onset. MRI volume data were segmented with Freesurfer and transformed in Montreal Neurological Institute space. A template source grid covering the entire brain volume was created on the default anatomy (10-mm resolution) and projected to individual anatomies to be used for the individual source-reconstruction procedure. We computed individual MEG forward head models using the overlapping-sphere method (volume) and source imaging using dSPM (v. 2016, median eigenvalue) onto preprocessed data, all by using default Brainstorm parameters. We obtained 1673 source volumes (i.e., vertices), each composed of three orientations (*x*, *y*, and *z*). The procedure also included an empirical estimate of the variance of the noise at each MEG sensor, obtained from a 2-min empty-room recording done at the beginning of each scanning session. One participant was excluded from subsequent analyses (hence, *n* = 29) as we failed to detect proper auditory responses.

#### Spectral decomposition of the MEG data

For both channel-level and source-level MEG data, trial-by-trial time-frequency decomposition was conducted in a range of 100 frequencies, logarithmically spaced from 1 to 100 Hz. Morlet wavelet transform was applied to the data using the Brainstorm (MATLAB) function bst_timefreq with parameter Method = “morlet,” central frequency Morlet_Fc = 1, and time resolution Morlet_FwhmTc = 3.

#### Normalized power spectrum

For each vertex, trial, and participant, we estimated the power spectrum by time-averaging the spectrally decomposed power signals in the [0.5 to 16] second range. 1/*f* Aperiodic component was removed by *z*-scoring the data across voxels for each frequency. We hence obtained, for each frequency, the distribution of power across voxels, centered around zero.

#### Modeling of the spectral gradient

We fitted the source-reconstructed spatial distribution of the MEG power spectrum, both at the group and individual levels, for both the MEG experiment and resting-state datasets. We approximated the data by estimating coefficients *p*_1_ to *p*_6_ with a five-order polynomial function *p*(*v*) = *p*_1_*v*^5^ + *p*_2_*v*^4^ + *p*_3_*v*^3^ + *p*_4_*v*^2^ + *p*_5_v + *p*_6_ using the polyfit function in MATLAB (MATLAB 2018b), fitting across vertices the dominant frequency of each vertex *v* to its spatial coordinate, for each spatial dimension independently (the *x*, *y*, and *z* coordinates of the source-reconstructed data). We projected the fitted function *p*(*v*) in the three-dimensional (3D) space, with the polyval function. Moreover, we estimated the quality of fit by means of the adjusted *r*^2^, which determines the proportion of variance explained by the model, adjusted for the number of coefficients.

### Multivariate pattern analysis on channel-level MEG data

Multivariate pattern decoding analyses were conducted by capitalizing on the spatial patterns of the MEG power signal, i.e., spectrally decomposed and time-averaged (0.5 to 16 s, to exclude the stimulus-onset period), for each participant, each neural frequency and each regressor (degree of syncopation, groove ratings, and acoustic temporal modulation spectrum). We used a cross-validated multivariate linear decoding model to estimate the spatial MEG patterns $\hat{w}$ of a specific data associated with each stimulus characteristic *X*. For each cross-validation fold [*n* = 10, interleaved; see (*79*)], we defined the spatial MEG patterns $\hat{w}$ on the training set by regressing, in a ridge sense (ridge α parameter set at 2), each *z*-scored MEG feature *Z*_*t*rain_ (248 channels in total) against the stimulus characteristic *X*_train_ across stimulus exemplars (*n* = 130 for each cross-validation fold), by solving $\hat{w}={\left(Z_{\text{train}}^{T}*Z_{\text{train}}+\alpha *I_{p}\right)}^{-1}*Z_{\text{train}}^{T}*X_{\text{train}}$ , where *I_p_* is the p*p identity matrix with *p* corresponding to the number of MEG channels (248 in total). We then projected the MEG data on the test set *Z*_test_, on the dimension defined by the coding weights $\hat{w}$ to obtain neural predictions of the stimulus characteristic *X*_test_ for each epoch of the test set (*n* = 14 for each cross-validation fold). After applying this procedure for each cross-validation fold, we computed the linear Pearson’s correlation coefficient between neural predictions $\hat{X}$ and ground-truth values *X* of the stimulus characteristic. The coding precision metric reported in the results section corresponds to the Fisher transform of the correlation coefficient, which is approximately normally distributed, such that we could compute standard parametric statistics at the group level.

### Searchlight analysis on source-level MEG data

We conducted searchlight-based multivariate pattern analyses on the entire power signal of the reconstructed volume sources (vertices), for each participant and each regressor (degree of syncopation and groove ratings). The searchlight procedure was applied to each vertex position by using as features the entire power spectrum of the current vertex and its 50 closest neighbors (in terms of Euclidean distance). Similarly, we estimated the coding of groove ratings at the source-level for three specific frequency bands, namely, beat-related (2 Hz), delta (1.3 to 1.5 Hz), and beta (20 to 30 Hz) neural dynamics.

#### Multivariate pattern analysis on ROIs

We defined five ROIs of 20 vertices each based on the contrast between the decoding of degree of syncopation and groove ratings at the source-level (Fig. 3G). The 20 vertices surrounding (in terms of Euclidean distance) the maximally significant vertex were selected. We then conducted multivariate pattern analyses on the power signal, for each participant, each frequency, each regressor, and each ROI.

#### Phase-amplitude coupling

We estimated phase-amplitude coupling over time (0.5 to 16 s) and melodies for each source-reconstructed vertex, between the phase at 1.4 or 2 Hz and the amplitude between 3 and 45 Hz following the procedure described in (*80*). In brief, from the spectral decomposition analysis (see above) we extracted the time-resolved phase ϕ of the 1.4- and 2-Hz activity. Simultaneously, we extracted the time-resolved amplitude signal ɑ of each investigated frequency (3 to 45 Hz). Then, we computed the phase-amplitude coupling ρ, estimated over *t* (time and melodies concatenated), for each combination of phase ϕ and amplitude ɑ frequencies

$$\rho =\frac{|\sum_{t=1}^{N}a_{t}.e^{i.\varphi_{t}}|}{\sqrt{N}.\sqrt{\sum_{t=1}^{N}a_{t}^{2}}}$$

#### Statistical procedures

All analyses were performed at the single-subject level and followed by standard parametric tests at the group level (e.g., two-tailed paired *t* tests, two-tailed *t* tests against zero, and rm-ANOVAs). The type 1 error rate arising from multiple comparisons was controlled for using FDR correction over the dimensions of interest (i.e., time, vertices, and frequencies), using the procedure introduced by Storey (*81*).

#### Neural network model

We implemented a canonical model for gradient frequency neural networks based on the GrFNN Toolbox [for more information, please see on musicdynamicslab.uconn.edu/home/multimedia/grfnn-toolbox/ and (*44**–**46*, *52*, *53*, *74*)]. The aim of this modeling approach was twofold. We first investigated whether neural resonance can explain the experience of groove during music listening and, specifically, whether a neurodynamic model can capture the nonlinear relationship between degree of syncopation and groove ratings (Fig. 2, B and C). Our secondary goal was to understand which neurodynamic mechanisms may underlie the experience of groove.

Our model is composed of three 1D networks of nonlinear oscillators, tuned to different natural frequencies. Such networks are conceptually like banks of band-pass filters, except that they consist of nonlinear oscillators rather than linear resonators. Network elements are canonical Hopf oscillators, a fully expanded canonical model for excitation-inhibition oscillations near a Andronov-Hopf bifurcation (*45*, *46*, *82*). As a generic model of excitation-inhibition oscillations, the canonical model represents the firing rates of interacting excitatory and inhibitory neural subpopulations as sinusoidal oscillations in the complex plane. The oscillators of each network are tuned to a range of distinct frequencies spanning the delta-theta frequency range and stimulated with time-varying acoustic signals given by

$${\overset{\cdot }{z}}_{i}=z_{i}\left(a_{i}+b_{i}{|z_{i}|}^{2}+\frac{d_{i}{|z_{i}|}^{4}}{1-{|z_{i}|}^{2}}\right)+x_{i}$$

where *z_i_* is the complex-valued state of the *i*th oscillator in the network (subscript *i* = 1,…, *N*), *a_i_* = α*_i_* + *i*ω*_i_*, *b_i_* = β_1*i*_ + *i*δ_1*i*_, d*_i_* = β_2*i*_ + *i*δ_2*i*_(α*_i_*, ω*_i_*, β_1*i*_, δ_1*i*_, β_2*i*_, δ_2*i*_ ∈ *R*; *i* denotes the imagery unit), and *x_i_* is the sum of input terms. The parameters α*_i_*, β_1*i*_ and β_2*i*_ determine the intrinsic dynamics of the *i*th oscillator, where α*_i_* is the bifurcation parameter; ω*_i_* is its natural frequency; δ_1*i*_ and δ_2*i*_ determine the dependence of intrinsic frequency on amplitude. The input to the *i*th oscillator *x_i_* can include both an external signal *s_i_*(*t*) corresponding to the onsets of rhythmic auditory stimuli and coupling from other oscillators

$$x_{i}=s_{i}\left(t\right)+\sum_{j\neq i}^{N}c_{\mathrm{ij}}z_{j}^{k}{\bar{z}}_{i}^{m-1}$$

where *c_ij_* is the coupling coefficient (*46*) and *k*:*m* is the mode-locking ratio (i.e., 1:1, 2:1, and 3:1).

For the first (auditory) layer, we set the parameters as α = 0.0001, β_1_ = 0, and β_2_ = −3. Thus, each oscillator in this network produces a low-amplitude intrinsic oscillation at its own intrinsic frequency that can be entrained by an external rhythmic stimulus (*52*, *83*, *84*). The second (motor planning) layer parameters were chosen as α = −0.8, β_1_ = 4, and β_2_ = −3. In this parameter regime, each oscillator was bistable (or double limit cycle) regime, exhibiting both a stable limit cycle at zero and a stable limit cycle at a higher amplitude. Oscillators in this network begin at rest, and, when it receives a signal that is strong enough and long enough, it can jump to a stable limit cycle. The third (groove) layer has the same parameters as layer two. The remaining parameters were all set to zero: δ_1_ = 0 and δ_2_ = 0. All the layers were based on 321 oscillators of internal frequency ω*_i_*, which span a range between 0.375 and 12 Hz (inclusive of the delta-theta frequency range) according to a log scale.

In this network, connections are learned according to the Hebbian rule (*74*, *85*)

$$\tau_{\mathrm{ij}}{\overset{\cdot }{c}}_{\text{ij}}=c_{\mathrm{ij}}\left(\lambda +\mu_{1}{|c_{\mathrm{ij}}|}^{2}+\frac{\mu_{2}{|c_{\mathrm{ij}}|}^{4}}{1-{|c_{\mathrm{ij}}|}^{2}}\right)+\kappa z_{i}^{m}{\bar{z}}_{j}^{k}$$

To make the dynamical interactions as transparent as possible, we designed a feedforward model with a minimal connection topology. Layer 1 is connected only to the auditory input, represented by the onset of the notes in the stimuli. We assumed learned multifrequency connections (black arrows in Fig. 2D) between layers 1 and 2. Initial connection strengths were chosen such that each frequency is connected to its one-fourth, one-third, one-half, first, second, third, and fourth harmonics. We then fixed the learning parameters as λ = −1, μ_1_ = 4, μ_2_ = −2.2, and κ = 0.2 (*74*). We then assumed fixed 1:1 excitatory connections from layers 2 and 3 with a coupling strength fixed as *w* = 0.8 and fixed 1:1 inhibitory connections from layers 1 to 3 (red arrow in Fig. 2D) with a coupling strength fixed as *w* = −0.7. Thus, oscillators in layer 3 received in-phase stimulation from the motor planning network and anti-phase stimulation from the auditory network. This means that the input to layer 3 was the difference between the time-dependent oscillations of layer 2 (pulse/meter) and layer 1 (auditory rhythm).

We ran the model as follows: The MIDI representation of each melody was used to provide a clear estimate of the note onsets. Note onsets were encoded as a sequence of continuous time onset pulses and transformed into a complex-valued signal using a Hilbert transform (a complex input is more relevant for this model, but real-valued signals can also be used). The model entrained to the onsets for the entire duration of the melodies (16 s). As with the data from the human experiment, we removed the initial (evoked) response of the model (here, the first 2 s). We then computed the mean field of each network individually [the mean of all oscillators in the network; see (*44*)]. Last, we computed fast fourier transform of the mean field (for 2 to 16 s of the stimulus) and extracted the amplitude of the response at 2 Hz for each layer of the model, for each melody. We repeated this procedure 29 times (similar to the number of participants in our MEG experiment) to obtain a robust estimate of the model’s response. Of note, the network dynamics were deterministic, but initial conditions were chosen at random, so the network output for each run of the same stimulus differed somewhat. Overall, this procedure produced an estimate of the amplitude of the time-averaged 2-Hz oscillations in each of the three network layers, in response to each of the 36 melodies.
