# Supplementary material for: Neural dynamics of predictive timing and motor engagement in music listening
Source: Sci Adv. 2024 Mar 6;10(10):eadi2525. doi: 10.1126/sciadv.adi2525 (PMC10917349; doi:10.1126/sciadv.adi2525)
Supplement: Supplementary file 1 — Supplementary Text Figs. S1 to S4 [file sciadv.adi2525_sm.pdf]

Science Advances
AAAS

Supplementary Materials for

Neural dynamics of predictive timing and motor engagement in music listening

Arnaud Zalta *et al.*

Corresponding author: Arnaud Zalta, arnaud.zalta@hotmail.fr; Benjamin Morillon, bnmorillon@gmail.com

Sci. Adv. **10**, eadi2525 (2024)
DOI: 10.1126/sciadv.adi2525

This PDF file includes:

Supplementary Text
Figs. S1 to S4

Supplementary Text

Supplementary Results

Modelling the relation between groove ratings and degree of syncopation. We fitted at the individual level the relation between groove ratings and degree of syncopation. We fitted both a linear and quadratic model, and compared their goodness-of-fit, by using the adjusted r-squared values. For the online experiment (n = 66), we obtained an average adjusted r-squared of 0.14 and 0.37 across melodies, for the linear and quadratic functions. The quadratic model significantly outperformed the linear one (t(65) = 13.1; $p < 0.001$). For the MEG experiment (n = 29), we obtained an average adjusted r-squared of 0.21 and 0.49 across melodies, for the linear and quadratic functions. The quadratic model significantly outperformed the linear one (t(28) = 11.8; $p < 0.001$). At the group level, the inverse U-shape profile is very well approximated with a quadratic function for both the online (adjusted $r^2(33) = 0.73$) and MEG (adjusted $r^2(33) = 0.67$) experiments.

Correlation of the degree of syncopation and groove ratings with the neural network model outputs. We first correlated the degree of syncopation with the time-averaged 2 Hz amplitude of the dynamics of each layer of the neurodynamic model. Layer 1 strongly linearly correlates with the degree of syncopation ($r^2(34) = 0.85$; $p < 0.001$), which is not the case for layer 2 ($r^2(34) = 0.12$; $p = 0.04$) or layer 3 ($r^2(34) = 0.14$; $p = 0.02$). Second, we correlated groove ratings, averaged across participants, obtained either from the online (n = 66) or the MEG (n = 29) experiment. Groove ratings mostly linearly correlated with activity from layer 3 (online: $r^2(34) = 0.66$; $p < 0.001$; MEG: $r^2(34) = 0.57$; $p < 0.001$), but to a far less extent with activity from layer 1 (online: $r^2(34) = 0.34$; $p < 0.001$; MEG: $r^2(34) = 0.38$; $p < 0.001$) or layer 2 (online: $r^2(34) = 0.19$; $p = 0.008$; MEG: $r^2(34) = 0.13$; $p = 0.03$). Our results show that a high groove experience reflects strong resonance in layer 2 (pulse/meter) at frequencies that are weak and/or anti-phase in the stimulus (and in layer 1), while difficulty or inability to perceive a pulse (the absence of *expectancy* in layer 2) is characteristic of high syncopated melodies (Fig. S1b).

Supplementary figures and legends

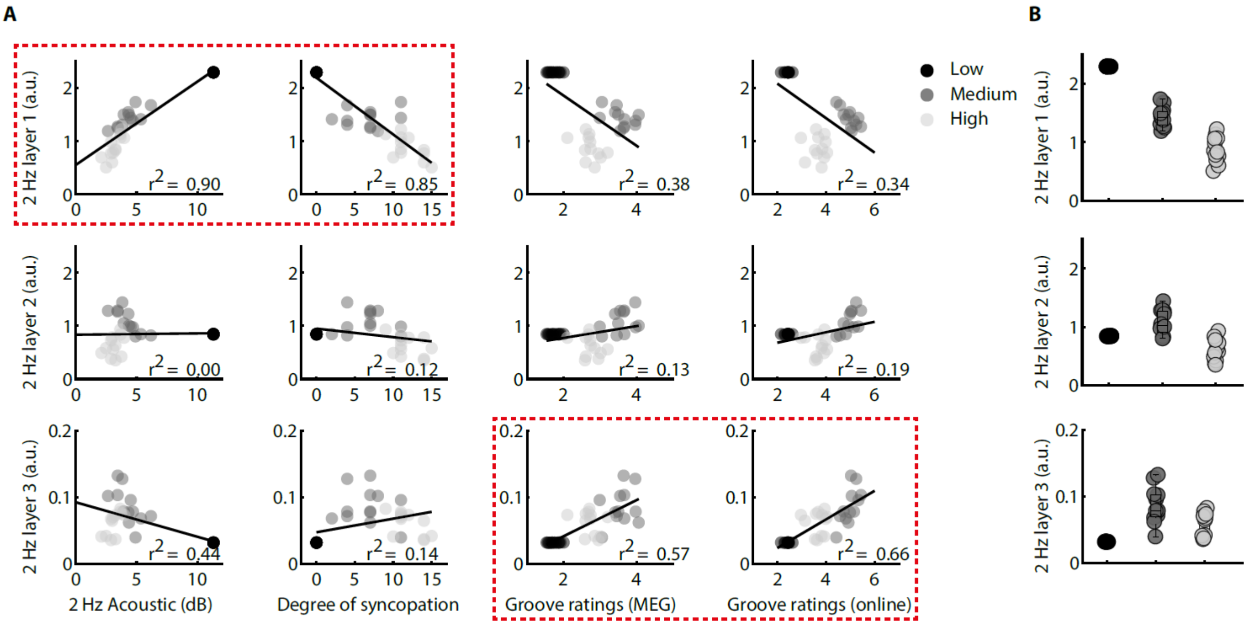

Figure S1. Neurodynamic model. (a) Inter-melody correlation between the time-averaged 2 Hz amplitude of each layer of the neurodynamical model (1 to 3; lines, y-axes), as a function of 4 paradigmatic variables (2 Hz acoustic, degree of syncopation, MEG groove ratings, or online groove ratings; columns, x-axes). Data were approximated with a linear function. Pearson's r-squared is reported. Strongest correlations are highlighted with red rectangles. **(b)** Amplitude of the time-averaged 2 Hz oscillations in the three network layers, in response to each of the 36 melodies, for each condition (low, medium, high). Shades of gray indicate the conditions. Individual points indicate melodies (n = 36).

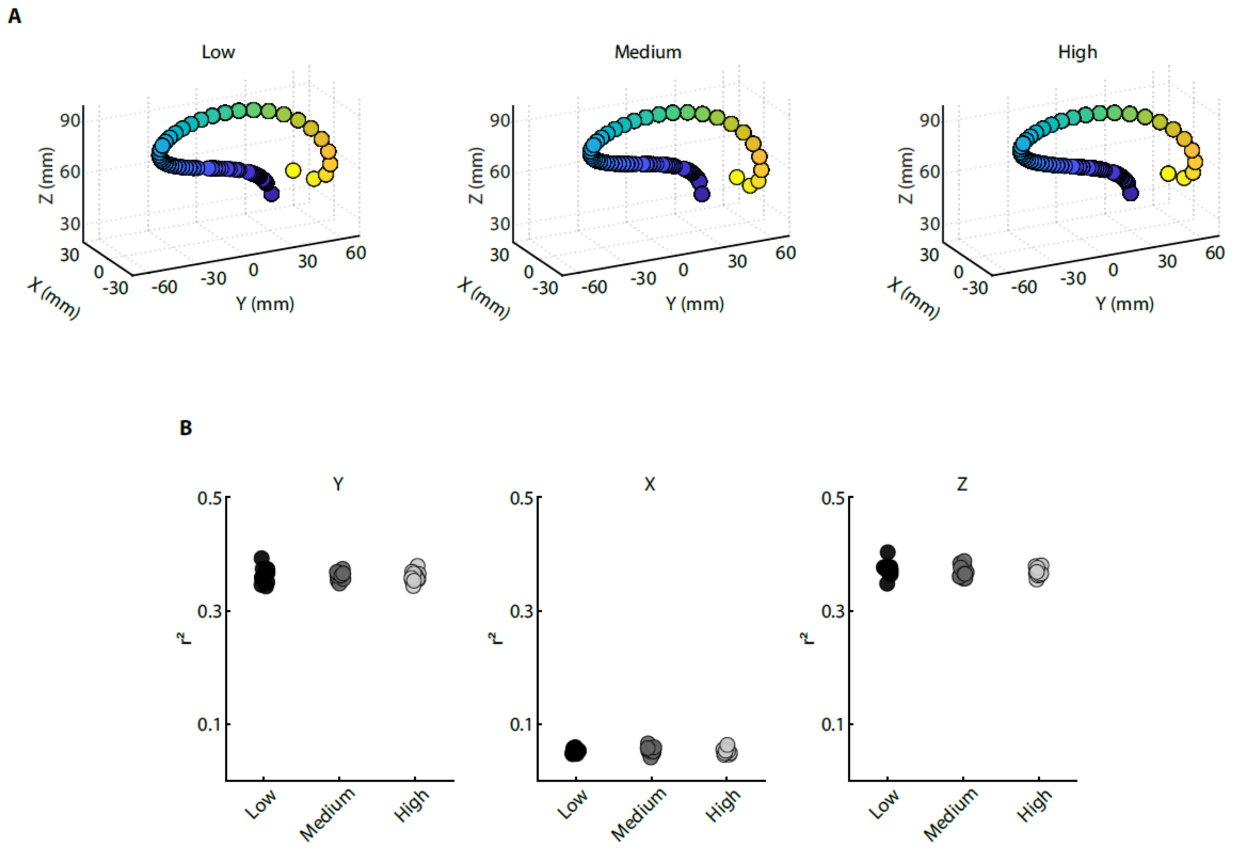

Figure S2. Dominant frequency across the brain volume in the three melodic conditions (low, medium, high). Frequency range 1-45 Hz (after removal of the 1/f decay of the neural power spectrum). **(a)** For each condition, data were approximated at the group-level with a polynomial function, independently for each dimension (X, Y, Z) of the MNI space. **(b)** Comparison of the quality of fits (r^2), estimated at the individual level, of the 36 melodies grouped per condition (low, medium, high) and plotted for each dimension (Y, X, Z) of the MNI space.

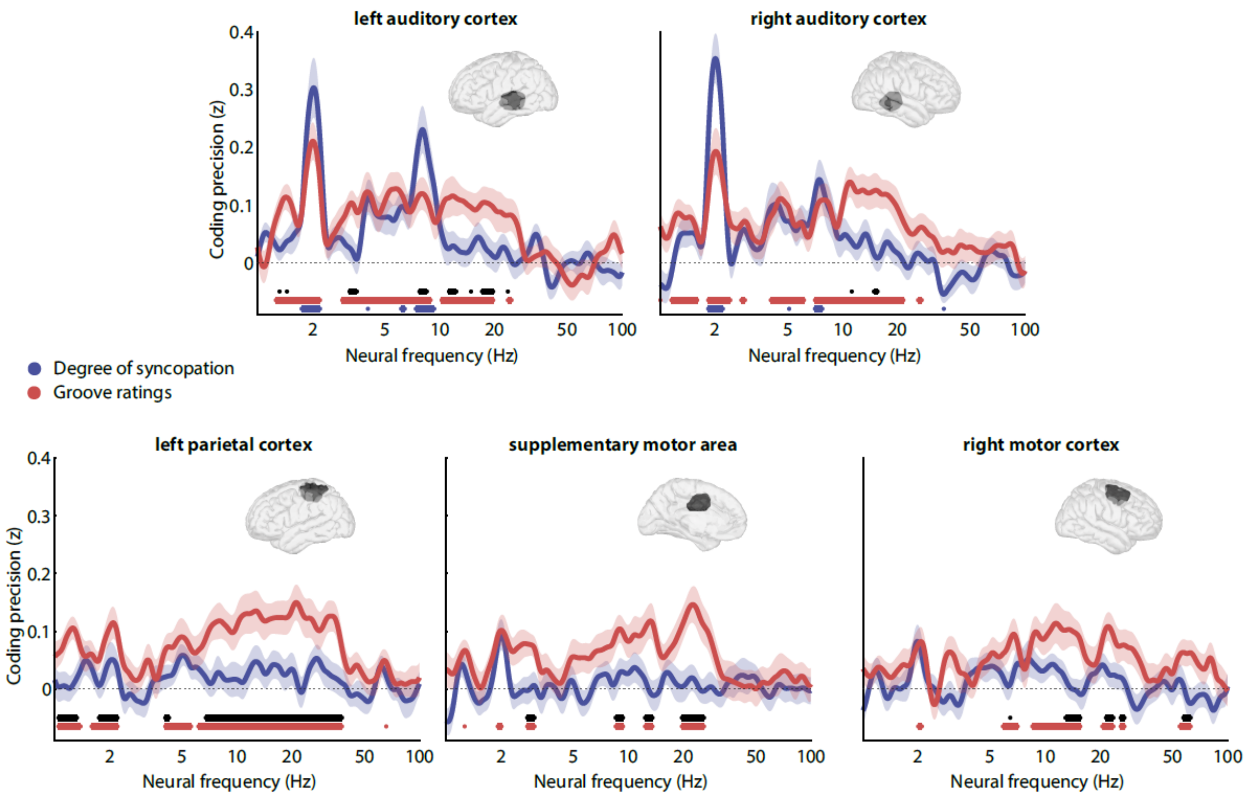

Figure S3. MEG experiment: Spectral coding of degree of syncopation and groove ratings in the regions-of-interest. Spectrum of neural coding of the degree of syncopation (blue) and groove ratings (blue), for each of the regions-of-interest (Fig. 4c). Red and blue horizontal lines indicate frequencies with significant coding values ($q < 0.005$, FDR-corrected). The black line indicates frequencies with significant differences in coding precision between degree of syncopation and groove ratings ($q < 0.05$, FDR-corrected). Error bars indicate SEM. Inset brains indicate the spatial localization of each region-of-interest.

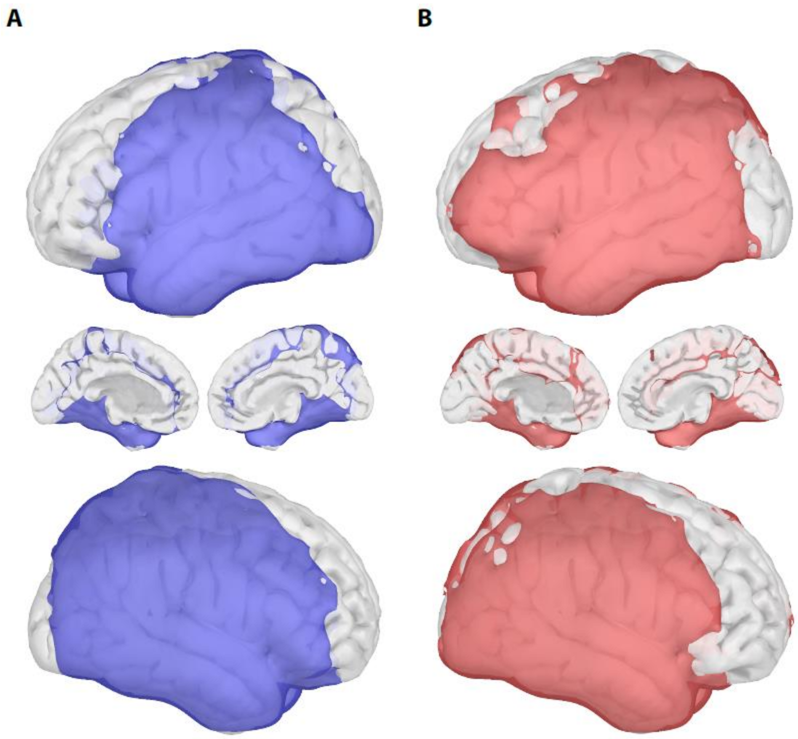

Figure S4. Spatial map of neural coding of (a) the degree of syncopation and (b) groove ratings from 2 Hz neural dynamics. Significant results reported at q < 0.005, FDR-corrected.
